# Association of Polymorphisms in three pri-miRNAs that Target Pepsinogen C with the Risk and Prognosis of Gastric Cancer

**DOI:** 10.1038/srep39528

**Published:** 2017-01-09

**Authors:** Ye-feng Wu, Qian Xu, Cai-yun He, Ying Li, Jing-wei Liu, Na Deng, Li-ping Sun, Yuan Yuan

**Affiliations:** 1 Tumor Etiology and Screening Department of Cancer Institute and General Surgery, the First Affiliated Hospital of China Medical University, and Key Laboratory of Cancer Etiology and Prevention (China Medical University), Liaoning Provincial Education Department, Shenyang 110001, People’s Republic of China; 2 Central Laboratory, Cancer Hospital of China Medical University, Liaoning Cancer Hospital & Institute, Shenyang 110042, People’s Republic of China; 3 Department of Molecular Diagnostics, Sun Yat-Sen University Cancer Center, State Key Laboratory of Oncology in South China, Collaborative Innovation Center for Cancer Medicine, Guangzhou, China; 4 Department of Oncology, The Fourth Affiliated Hospital of China Medical University, Liaoning, Shenyang 110032, People’s Republic of China

## Abstract

We aimed to explore the associations of polymorphisms in three microRNAs (miRNAs) (let-7e rs8111742, miR-365b rs121224 and miR-4795 rs1002765) that target PGC with the risk and prognosis of gastric cancer/atrophic gastritis. Sequenom’s MassArray was used to genotype the miRNA polymorphisms in 724 gastric cancer cases, 862 atrophic gastritis cases and 862 controls in a Chinese population. We found that let-7e rs8111742 and miR-4795 rs1002765 were associated with the risk of gastric cancer in the *H. pylori*-positive subgroup. MiR-365b rs121224 was associated with the risk of intestinal-type gastric cancer in the alcohol consumption subgroup. Intestinal-type gastric cancer patients at Borrmann stages III-IV who carry the miR-365b rs121224 GG genotype had better prognosis compared with those who carry the CG or CC genotypes. MiR-365b rs121224 was associated with Lauren typing and TNM staging, in which the distribution of GG genotype carriers in intestinal-type gastric cancer and the TNM stage I-II subgroup was higher than that of CG or CC genotypes, which contrasted with the distribution in diffuse-type gastric cancer or TNM III-IV groups. These findings suggested that the polymorphisms in these miRNAs might be biomarkers for gastric cancer risk and prognosis, especially for populations infected with *Helicobacter pylori* or who consume alcohol.

Gene polymorphisms are a common genetic variant. The most common polymorphic form is a base difference, termed a single nucleotide polymorphism (SNP). Approximately 50% of SNPs occur in the noncoding region of a gene. MicroRNAs (miRNAs) are single stranded, 18–23 nucleotide-long, RNA molecules, which can be polymorphic and can affect maturation and function of the miRNA, possibly resulting in disease. For example, the pri-miR-15a/miR-16 C/T polymorphism is associated with familial chronic lymphocytic leukemia^1^; pri-miR-218 rs11134527 is associated with the risk of liver cancer in the Chinese population^2^; pri-miR-185 rs2008591 is associated with the risk of breast cancer^3^; pre-miR-423 rs6505162 and pre-miR-608 rs4919510 are associated with the prognosis of colorectal cancer^4^; miR-146a G/C and pri-let-7a-2 rs629367 are associated with the prognosis of gastric cancer^5^,^6^; pre-miR-196a2 rs11614913 and pre-miR-146a rs2910164 are associated with the prognosis of non-small cell lung cancer^7^,^8^; and pre-miR-146a rs2910164 is associated with the prognosis of adult gliomas^9^. Although there are some studies on the association of the risk and prognosis of cancer with single polymorphic miRNAs, there are few reports on the relationship between target gene-based miRNA polymorphisms and cancer. MiRNAs cause mRNA degradation or translational containment through complete or incomplete complementarity with the 3′ non-coding sequence of its target gene^10^. Polymorphic miRNAs can affect the process of miRNA maturation, and then affect its regulatory function on the target gene^1^,^2^,^3^. Single miRNAs can regulate different genes and multiple miRNAs can be regulated by the same target gene. The exploration of the relationship between targeted gene-based miRNA polymorphisms and cancer would be helpful to discover the potential of miRNA target gene-related diseases. It would also be helpful to clarify the role of miRNA genetic variation and its target gene dysregulation in cancer development, including determining the molecular pathways of miRNAs involved in target gene regulation.

Pepsinogen C (encoded by *PGC*) is the mature form of an aspartic proteinase present in the gastric mucosa and serum, and is used in a serological test for early screening of gastric cancer and precancerous disease. Our previous studies in normal gastric mucosa, atrophic gastritis (precancerous disease group) and the gastric disease chain showed that PGC protein levels decrease gradually with disease progression^11^. The serological detection of PGC can be used to evaluate and manage the advance of gastric cancer and precancerous diseases^12^. However, the regulation of the PGC protein levels is not clear.

Previously, we used the NCBI bioinformatics database to predict miRNAs that might target *PGC*, and identified let-7e, miR-4795 and miR-365b, which were confirmed by luciferase reporter experiments. According to the screening rule that a polymorphic locus in the Chinese population should exist with a frequency distribution and minor allele frequency (MAF) > 5, we screened three tagSNPs (SNPs that uniquely represent a haplotype) located in the primary precursor regions of the three miRNAs. They were, pri-let-7e rs8111742, located in the let-7e gene promoter region at −565 bp; pri-miR-365b rs121224, located in the miR-365b gene promoter region at −430 bp; and pri-miR-4795 rs1002765, located in pri-miR-4795 gene promoter region at −1635 bp. These three tagSNPs are all located in important promoter regions. Whether these miRNA SNPs are associated with the downregulation of *PGC* in atrophic gastritis or gastric cancer; whether they can be used as diagnostic or prognostic markers of gastric cancer; and what are the relationships between miRNA polymorphisms and *Helicobacter pylori* infection, smoking and alcohol consumption, as well as other gastric cancer environmental factors, remain unclear.

This case-control study aimed to explore the relationships between polymorphisms in the *PGC*-targeting miRNAs, pri-let-7e, pri-miR-365b, pri-miR-4795, with the risk and prognosis of atrophy gastritis and gastric cancer in a northern Chinese population to explore their potential as specific markers of gastric cancer and its precursor. This study could provide a theoretical and experimental basis for further exploration of the genetic variation of these three miRNAs and the dysregulation of their target gene, *PGC*, in gastric cancer development.

## Results

### Subject information

The study subjects included 862 patients with atrophic gastritis, 724 with gastric cancer and 862 control subjects. There was no significant difference in the age or sex distribution between the gastric cancer group and the control group or between the atrophic gastritis group and the control group. The characteristics of the three groups are shown in Table 1. We selected 357 subjects who underwent surgical treatment and were subjected to regular follow-up. The prognosis of gastric cancer patients with complete clinical pathology data was studied. Clinical staging of gastric cancer used the seventh edition of the UICC TNM staging^13^, and Lauren typing^14^ was used for the histological classification of gastric cancer (Table 1).

### Association of pri-let-7e rs8111742, pri-miR-365b rs121224 and pri-miR-4795 rs1002765 with risk of atrophic gastritis and gastric cancer

The overall analysis of all cases showed no significant correlations for the three miRNA polymorphisms with the risk of atrophic gastritis or gastric cancer (Table 2).

### Stratified analysis of pri-let-7e rs8111742, pri-miR-365b rs121224 and pri-miR-4795 rs1002765 and risk of atrophic gastritis and gastric cancer

We further analyzed environmental factors such as *H. pylori* infection, smoking and alcohol consumption, and found that the let-7e rs8111742 AA genotype could increase the risk of gastric cancer (*P* = 0.044) in the *H. pylori*-positive group, while the miR-4795 rs1002765 AA genotype could reduce the risk of gastric cancer (*P* = 0.050) in the *H. pylori*-positive group (Table 3).

### Associations of pri-let-7e rs8111742, pri-miR-365b rs121224 and pri-miR-4795 rs1002765 with the risk of intestinal-type and diffuse-type gastric cancer

By Lauren typing, we divided the gastric cancer patients into intestinal-type and diffuse-type. The associations of the three miRNA polymorphisms with the risk of intestinal-type and diffuse-type gastric cancer were then analyzed. There were no significant correlations between any of the miRNA polymorphisms and the risk of either gastric cancer (Table 4). Further analysis of environmental factors, such as *H. pylori* infection, smoking and alcohol consumption, found that in the alcohol consumption subgroup, the pri-miR-365b rs121224 GG genotype could increase the risk of intestinal-type gastric cancer compared with the CC genotype (*P* = 0.029) and the CG + CC genotype (*P* = 0.045), respectively. For the pri-miR-4795 rs1002765 polymorphism, the GA + AA genotype could reduce the risk of diffuse-type gastric cancer compared with the GG genotype (*P* = 0.032). The pri-miR-4795 rs1002765 AA genotype could reduce the risk of diffuse-type gastric cancer in the *H. pylori* infection-positive subgroup compared with the GG and GA + GG genotypes (*P* = 0.005, respectively) (Table 5).

### Correlation of pri-let-7e rs8111742, pri-miR-365b rs121224 and pri-miR-4795 rs1002765 with clinical pathological parameters

MiR-365b rs121224 was related to Lauren and TNM staging. In contrast with diffuse-type gastric cancer or the TNM III-IV stage subgroup, the distribution frequency of GG genotype carriers in the intestinal-type gastric cancer subgroup and in the TNM I-II stage subgroup was higher than that of the CG + CC genotype carriers (*P* = 0.014, *P* = 0.031 respectively) (Table 6).

### Association of pri-let-7e rs8111742, pri-miR-365b rs121224 and pri-miR-4795 rs1002765 with gastric cancer prognosis

The miR-365b rs121224 GG genotype was associated with a better prognosis compared with the CG or CC genotypes in the Borrmann III-IV subgroup and in the intestinal-type gastric cancer subgroup (*P* = 0.042, *P* = 0.031, respectively) (Table 7).

## Discussion

This paper explored the associations of polymorphisms in three miRNAs (let-7e rs8111742 G > A, miR-365b rs121224 C > G and miR-4795 rs1002765 G > A) that target *PGC* with the risk and prognosis of gastric cancer and atrophic gastritis. Overall risk analysis revealed that there was no significant correlation between the three miRNA polymorphisms and gastric cancer or atrophic gastritis. However, subgroup analysis revealed that let-7e rs8111742 and miR-4795 rs1002765 were associated with the risk of gastric cancer in the *H. pylori*-positive subgroup. MiR-365b rs121224 was associated with intestinal-type gastric cancer risk in the alcohol consumption subgroup, and miR-4795 rs1002765 was associated with diffuse-type gastric cancer risk in the *H. pylori*-positive and alcohol consumption subgroups. Prognostic analysis revealed that intestinal-type gastric cancer patients at Borrmann stage III-IV who carry miR-365b rs121224 GG genotype have a better prognosis compared with those who carry the CG or CC genotypes. MiR-365b rs121224 was associated with Lauren typing and TNM staging, in which the distribution of GG genotype carriers in intestinal-type gastric cancer and the TNM stage I-II subgroup was higher than that of CG or CC genotypes, which is in contrast with the distribution in diffuse-type gastric cancer or TNM III-IV groups. To the best of our knowledge this is the first study to report on the association of three miRNA tagSNPs that target *PGC* with the risk and prognosis of gastric cancer and atrophic gastritis in a Chinese population.

MiRNA polymorphisms may be associated with susceptibility to disease^15^. Certain miRNA polymorphisms are associated with the overall population risk, some of which are associated with risk in a specific population. In this study, we did not find that let-7e rs8111742 G > A, miR-365b rs121224 C > G and miR-4795 rs1002765 G > A are associated with gastric cancer and atrophic gastritis risk in the general population. However, in the subgroup analysis, it was found that the three miRNA polymorphisms were related to a specific population who are infected with *H. pylori* or who consume alcohol. *H. pylori* is one of the most important environmental factors in the stomach, and the development of intestinal-type gastric cancer and diffuse-type gastric cancer are related to *H. pylori* infection^11^. This study found that the let-7e rs8111742 and miR-4795 rs1002765 polymorphisms increased and decreased, respectively, the risk of gastric cancer in the *H. pylori*-positive subgroup. This indicated that the let-7e mutation might be a risk genotype, which might have a positive effect on the occurrence and development of gastric cancer, and that the miR-4795 mutation may play a protective role in the development of gastric cancer. Furthermore, *H. pylori* may be an intermediary or bridge that mediates host gene polymorphisms and susceptibility to gastric cancer. Although there are no other reports on the relationship between these two polymorphisms and disease, studies have found that other miRNA polymorphisms are associated with gastric disease in *H. pylori* infected patients. For example, the interaction between *H. pylori* and the TLR4 gene and miR-146a polymorphisms has been studied. It was found that the combined effect of the miR-146a rs2910164 GG genotype and the TLR4 + 3725C allele could increase the risk of severe atrophic gastritis in a Japanese population infected with *H. pylori*^16^. In addition, Song *et al*. found that in an *H. pylori*-positive subgroup, the miR-146a rs2910164 C allele increased the risk of intestinal metaplasia and dysplasia^17^. Okubo *et al*. found that the rs11614913 TT polymorphism in miR-196a-2 correlated positively with the degree of induced monocyte infiltration in *H. pylori* infection^18^. These findings suggest that a high risk miRNA polymorphism carrier with *H. pylori* infection is at higher risk of suffering from gastric cancer and precancerous disease; therefore, more attention should be paid to their follow-up and to individual prevention. Similarly, our study found that miR-365b rs121224 was associated with intestinal-type gastric cancer risk in the alcohol consumption subgroup, and miR-4795 rs1002765 was associated with diffuse-type gastric cancer risk in the *H. pylori*-positive and alcohol consumption subgroup. Thus, a high risk miRNA polymorphism carrier that drinks alcohol also has a potential risk of suffering from gastric cancer and precancerous disease, and should be vigilant.

In the prognosis analysis, we found that the GG genotype of miR-365b rs121224 was associated with better prognosis in the Borrmann III-IV and intestinal-type gastric cancer subgroups. There are two copies of the miR-365 gene in the human genome. MiR-365a is located on chromosome 16 and miR-365b is on chromosome 17; however, they both produce the same mature miR-365. The rs121224 polymorphism is located 430 bp upstream of miR-365b in the promoter region. The biological function of miR-365 is uncertain. Some scholars believe that miR-365 is an “oncogene”. It is expressed at high levels in skin squamous cell carcinoma and in some cancer cell lines, and *in vivo* experiments confirmed that over-expression of mir-365 can promote subcutaneous tumors in mice^19^. Furthermore, inhibition of mir-365 expression can cause cells to arrest in G1 phase and undergo apoptosis, thus inhibiting the formation of skin tumors^20^,^21^. However, in recent years, mir-365 has been shown to inhibit cancer. It is downregulated in lung cancer tissues; inhibits tumor cell line migration^22^,^23^,^24^,^25^,^26^,^27^; and promotes apoptosis and regulates bcl-2 expression^23^,^24^. The results of this study showed that the risk of intestinal-type gastric cancer in the alcohol consumption subgroup was lower with the pri-miR-365b rs121224 C allele, but that the subgroup of patients with Borrmann type III-IV staging had a better prognosis with the G genotype. The transcription factors Sp1 and NF-κB are associated with the promoter region of miR-365 and they activate cellular signaling pathways^22^; therefore, the genotype of rs121224 may affect the involvement of miR-365 in cell signaling pathways. Upregulation of miR-365 can also lead to changes in the Akt/PTEN/p53 pathway, with downregulation of PTEN protein levels^27^ leading to the accumulation of alcohol in cells^28^. Thus, the polymorphism in the miR-365 promoter region may be involved in the pathogenesis of intestinal-type gastric cancer by altering cell signaling. SNP rs121224 is located in the precursor region of pri-miR-365b and can affect the expression level and maturation process of the miRNA, which may affect its function. We hypothesized that the rs121224 polymorphism in the promoter region might be associated with decreased binding capacity of miR-365 transcription factors (Sp1 and NF-κB) and thus it is not able to stimulate cell proliferation via the MAPK pathway; thus the carriers have better prognosis. In gastric cancer progression, we found that the frequency of rs121224 GG genotype carriers in the intestinal-type gastric cancer subgroup and the TNM stage I-II subgroup was significantly higher than that of CG + CC carriers, which is consistent with the Borrmann type III-IV stage subgroup of patients, who had better prognosis with the G genotype.

MiRNAs let-7e, miR-4795 and miR-365b all target PGC. As we know PGC is a product of terminally differentiated gastric mucosa. The expression of PGC protein decreased gradually with gastric disease progression^11^. Our previous studies showed that a *PGC* polymorphism was also associated with gastric cancer and atrophic gastritis^29^,^30^,^31^. It is well accepted that gene–gene interactions are more important than single genes in promoting cancer susceptibility^32^,^33^,^34^. For example, gene polymorphisms that individually have a weak effect can have a strong effect when acting in synergy^35^. Similarly, the epistatic effect, which is a phenomenon that consists of the effect of complex interactions, is greater than the main effects of any single susceptibility gene^31^,^34^. The latest research in our group found that when these three miRNA polymorphisms and their target gene PGC polymorphisms are present together, epistasis occurs and the SNP–SNP interaction between the three miRNAs and their target *PGC* can increase the prediction risk of atrophic gastritis from 1.49 to 6.95 times^36^. Beside epistatic effect, it should be noted that miRNAs can affect the expression of its target genes by binding to 3′UTR regions. Thus, genetic variation of miRNA may be involved in the regulation of its target gene expression. Several studies have demonstrated that a variety of miRNAs may bind with PGC and affect its expression. For example, Liu *et al*. reported that serum let-7 microRNA negatively regulated the expression of PGC gene^37^. Other scholars suggested that miR-27a rs895819 polymorphism could affect the expression of its targeted gene ZBTB10^38^. And the promoter polymorphism of miR-34b/c rs4938723 could influence the transcription activity of miR-34b/c promoter, which therefore affect miRNA expression^39^,^40^. As the three described polymorphisms of let-7e, miR-4795 and miR-365b are all located in the important promoter regions, we speculate that these miRNA SNPs may be associated with the downregulation of PGC expression, thus increasing gastric cancer risk. Further study would be warranted to verify our assumptions and determine the molecular pathways of the miRNA polymorphisms involved in the regulation of the targeted PGC gene.

Several limitations of our study should be noted. First, the number of cases needs to be expanded to enable a stratified analysis. Second, data from other environmental factors, such as diet, should be analyzed. Third, functional studies are needed to determine the pathogenic pathways in which the miRNA polymorphisms operate.

In summary, we performed a case-control study to explore the associations of polymorphisms in three miRNAs that target *PGC* with the risk and prognosis of gastric cancer/atrophic gastritis. We found that pri-let-7e rs8111742 in the *H. pylori* infection-positive subgroup was associated with the risk of gastric cancer and that miR-4795 rs1002765 in the *H. pylori* infection-positive and alcohol consumption subgroups was associated with diffuse-type gastric cancer. Pri-miR-365b rs121224 is associated with intestinal-type gastric cancer in the alcohol consumption subgroup, and the G allele was found to have a better prognosis in patients with Borrmann III–IV staging and intestinal-type gastric cancer. These findings suggest that these miRNA polymorphisms may be markers for gastric cancer risk and prognosis, especially related to specific populations infected with *H. pylori* or who consume alcohol. This study also provided an experimental basis for further study of the regulation of PGC in the pathogenesis of gastric cancer.

## Materials and Methods

### Patients

This study was approved by the ethics committee of the First Affiliated Hospital of China Medical University. Written informed consent was obtained from all participants. We confirm that all experiments were performed in accordance with relevant guidelines and regulations. We enrolled 2448 subjects, including 862 atrophic gastritis, 724 gastric cancer and 862 control cases. The control group and atrophic gastritis group were all selected from the Zhuanghe Gastric Diseases Screening Program, which has been previously reported^12^. Fasting venous blood and biopsies were collected from subjects and histopathological diagnosis performed. The control group was confirmed to be normal or to have mild superficial gastritis by microscopic examination. Sydney classification^41^,^42^ was used to confirm atrophic gastritis, and patients with moderate to severe atrophic gastritis were selected for the atrophic gastritis group. Gastric cancer cases were all patients attending the First Affiliated Hospital of China Medical University. After admission, endoscopic biopsy and tissue pathology diagnosis were carried out. Patient data (including age, gender, smoking and drinking habits) were recorded and have been published previously^29^. For the prognosis study, we selected subjects who underwent surgical treatment and were subjected to regular follow-up; ultimately, 357 gastric cancer patients with complete clinical pathology data were studied. The clinical staging of gastric cancer used the seventh edition of UICC TNM staging^13^, and Lauren typing^14^ was used for the histological classification of gastric cancer.

### Subject’s genotyping

DNA was extracted from the patients’ venous blood, and Sequenom’s MassArray system was used to conduct polymorphism typing in all cases^29^. Repeated verification (Sequenom’s MassArray system) was performed for 10% of cases and the repetition rate was >99%.

### The determination of serum Helicobacter pylori -IgG titer

The serum *H. pylori*-IgG titer was detected using an enzyme-linked immunosorbent assay (Helicobacter pylori IgG kit; Biohit, Helsinki, Finland) according to a previously described method^5^,^43^. Patients with a serum titer >34 IU were diagnosed as *H. pylori* positive.

### Statistics

The dominant model compared heterozygotes and homozygous mutant with wild-types and the recessive model compared homozygous mutants with wild-types and heterozygotes. The distribution of demographic characteristics in the case and control groups, and the frequency distribution of genotypes in the disease group were measured using the χ^2^ test, and Student’s t test was used to assess age and other data. *P* < 0.05 was considered statistically significant.

## Additional Information

**How to cite this article:** Wu, Y.-f. *et al*. Association of Polymorphisms in three pri-miRNAs that Target Pepsinogen C with the Risk and Prognosis of Gastric Cancer. *Sci. Rep.* **7**, 39528; doi: 10.1038/srep39528 (2017).

**Publisher's note:** Springer Nature remains neutral with regard to jurisdictional claims in published maps and institutional affiliations.

## Figures and Tables

**Table 1:** The basic information of the research subjects for the risk and prognosis studies.

Variables	For the risk study				For the prognosis study			
	AG vs. CON		GC vs. CON		Gastric cancer	death	Median survival time (M)	*P*
	CON(%)	AG(%)	CON(%)	GC(%)				
	**n** = **862**	**N** = **862**	**n** = **729**	**n** = **724**	n = 357	n = 89		
Gender	*P* = 0.846		*P* = 0.564					
Male	483(56.0)	487(56.5)	483(66.3)	490(67.7)	255	63	60.8^a^	0.797
Female	379(44.0)	375(43.5)	246(33.7)	234(32.3)	102	26	45.7^a^	
Age	*P* = 0.343		*P* = 0.562					
Mean ± SD	54.9 ± 9.2	55.4 ± 9.5	56.1 ± 9.2	56.4 ± 9.8	/	/	/	
Median	54	56	56	57	/	/	/	
Range	17–85	16–79	17–85	21–81	/	/	/	
*H. pylori*	*P* < 0.001		*P* < 0.001					
Positive	241(28.0)	505(58.6)	201(27.6)	369(51.0)	/	/	/	
Negative	621(72.0)	41.4(36.5)	528(72.4)	355(49.0)	/	/	/	
Smoking	**n** = **586**	**N** = **548**	**n** = **500**	**n** = **333**				
	*P* = 0.299		*P* = 0.183					
Ever Smoker	202(34.5)	173(31.6)	199(39.8)	148(44.4)	/	/	/	
Never Smoker	384(65.5)	375(68.4)	301(60.2)	185(55.6)	/	/	/	
Drinking	**n** = **585**	**N** = **547**	**n** = **499**	**n** = **296**				
	*P* = 0.333		*P* = 0.044					
Drinker	147(25.1)	124(22.7)	146(29.3)	107(36.1)	/	/	/	
Nondrinker	438(74.9)	423(77.3)	353(70.7)	189(63.9)	/	/	/	
Borrmann type								0.055
Borrmann I–II	/	/	/	/	83	23	66.5^a^	
Borrmann III–IV	/	/	/	/	274	66	53.8^a^	
Lauren type								0.234
Intestinal type	/	/	/	/	120	25	59.9^a^	
Diffuse type	/	/	/	/	231	61	59.3^a^	
Non classified carcinoma	/	/	/	/	6			
TNM stage								9.40 × 10^−15^
I–II	/	/	/	/	171	12	73.5a	
III–IV	/	/	/	/	186	77	27	
Depth of invasion								5.35 × 10^−9^
T1 + T2	/	/	/	/	98	3	76.8^a^	
T3 + T4	/	/	/	/	259	86	52.2^a^	
Lymph node metastasis								1.82 × 10^−8^
positive	/	/	/	/	216	77	32	
negtive	/	/	/	/	141	12	71.5^a^	

^a^Mean survival time was provided when MST could not be calculated.

**Table 2:** Relationship between pri-let-7e rs8111742, pri-miR-365b rs121224, pri-miR-4795 rs1002765 and risk of atrophic gastritis and gastric cancer.

	Control group (%)	Atrophic gastritis group (%)	Atrophic gastritis group vs. control group		Control group (%)	Cancer group (%)	Cancer group vs. control group	
			*P*	OR(95%CI)			*P*	OR(95%CI)
	n = 862	n = 862			n = 729	n = 724		
**pri-let7e rs8111742**								
GG	459(53.2)	458(53.1)		1.00	389(53.4)	375(51.8)		1.00
GA	341(39.6)	343(39.8)	0.921	1.01(0.82–1.25)	285(39.1)	291(40.2)	0.690	1.05(0.84–1.31)
AA	62(7.2)	61(7.1)	0.987	1.00(0.67–1.48)	55(7.5)	58(8.0)	0.795	1.06(0.70–1.59)
AA + GA vs. GG			0.927	1.01(0.83–1.23)			0.667	1.05(0.85–1.30)
AA vs. GA + GG			0.984	1.00(0.68–1.47)			0.854	1.04(0.70–1.54)
A vs. G			0.929	1.01(0.86–1.18)			0.658	1.04(0.88–1.23)
HWP^a^	0.185	0.778			0.78	0.882		
**pri-miR-365b rs121224**								
CC	270(31.3)	272(31.6)		1.00	229(31.4)	221(30.5)		1.00
CG	428(49.7)	413(47.9)	0.436	0.91(0.73–1.15)	362(49.7)	376(51.9)	0.630	1.06(0.83–1.35)
GG	164(19.0)	177(20.5)	0.604	1.08(0.81–1.43)	138(18.9)	127(17.5)	0.806	0.96(0.70–1.32)
CG + GG vs. CC			0.695	0.96(0.77–1.19)			0.773	1.03(0.82–1.30)
GG vs. CG + CC			0.334	1.13(0.88–1.45)			0.593	0.93(0.71–1.22)
G vs. C			0.730	1.03(0.89–1.18)			0.963	1.00(0.86–1.16)
HWP^a^	0.8	0.3			0.811	0.129		
**pri-miR4795 rs1002765**								
GG	307(35.6)	304(35.3)		1.00	262(35.9)	272(37.6)		1.00
GA	420(48.7)	416(48.3)	0.766	1.03(0.83–1.29)	357(49.0)	349(48.2)	0.619	0.94(0.75–1.19)
AA	135(15.7)	142(16.5)	0.654	1.07(0.79–1.45)	110(15.1)	103(14.2)	0.703	0.94(0.67–1.30)
AA + GA vs. GG			0.694	1.04(0.85–1.28)			0.590	0.94(0.76–1.17)
AA vs. GA + GG			0.721	1.05(0.80–1.38)			0.774	0.96(0.71–1.29)
A vs. G			0.638	1.04(0.90–1.20)			0.609	0.96(0.82–1.12)
HWP^a^	0.662	0.988			0.518	0.597		

Note: ^a^HardyWeinberg balance in the crowd.

**Table 3:** Stratified analysis of pri-let-7e rs8111742, pri-miR-365b rs121224, pri-miR-4795 rs1002765 and risk of atrophic gastritis and gastric cancer.

Variables	Genotype	Atrophic gastritis group vs. control group	*P*	OR(95%CI)	Cancer group vs. control group	*P*	OR(95%CI)
**pri-let7e rs8111742**							
*H. pylori*^a^		n = 862 vs 862		n = 724 vs 729			
Negative	GG	193/329		1.00	196/278		1.00
	GA	139/245	0.814	0.97(0.74–1.27)	138/206	0.700	0.95(0.71–1.26)
	AA	25/47	0.685	0.90(0.54–1.51)	21/44	0.181	0.69(0.40–1.19)
	GA + AA VS. GG		0.746	0.96(0.74–1.24)		0.445	0.90(0.69–1.18)
	AA VS. GA + GG		0.737	0.92(0.55–1.52)		0.185	0.69(0.41–1.19)
Positive	GG	265/130		1.00	179/111		1.00
	GA	204/96	0.658	1.08(0.78–1.49)	153/79	0.316	1.21(0.84–1.74)
	AA	36/15	0.641	1.16(0.62–2.21)	37/11	**0.044**	**2.09(1.02–4.26)**
	GA + AA VS. GG		0.598	1.09(0.80–1.48)		0.118	1.32(0.93–1.87)
	AA VS. GA + GG		0.707	1.13(0.60–2.11)		0.064	1.94(0.96–3.89)
Smoking status^b^		n = 548 vs 586		n = 333 vs 500			
Smokers	GG	86/106		1.00	72/104		1.00
	GA	70/79	0.566	1.14(0.73–1.80)	65/78	0.416	1.22(0.76–1.96)
	AA	17/17	0.452	1.35(0.62–2.92)	11/17	0.754	0.87(0.35–2.14)
	GA + AA VS. GG		0.442	1.19(0.77–1.83)		0.519	1.16(0.74–1.84)
	AA VS. GA + GG		0.523	1.28(0.60–2.70)		0.590	0.78(0.32–1.92)
Non-smokers	GG	207/199		1.00	98/159		1.00
	GA	143/160	0.213	0.81(0.59–1.13)	74/122	0.500	0.87(0.58–1.31)
	AA	25/25	0.859	1.06(0.56–2.03)	13/20	0.974	1.01(0.46–2.22)
	GA + AA VS. GG		0.286	0.84(0.62–1.15)		0.535	0.88(0.60–1.31)
	AA VS. GA + GG		0.695	1.13(0.61–2.11)		0.838	1.08(0.50–2.33)
Drinking status^b^		n = 547 vs 585		n = 296 vs 499			
Drinkers	GG	60/74		1.00	48/73		1.00
	GA	49/63	0.884	0.96(0.57–1.63)	51/63	0.286	1.34(0.78–2.31)
	AA	15/10	0.161	1.87(0.78–4.51)	8/10	0.780	1.16(0.41–3.30)
	GA + AA VS. GG		0.736	1.09(0.66–1.79)		0.289	1.33(0.79–2.25)
	AA VS. GA + GG		0.136	1.94(0.81–4.61)		0.965	0.98(0.35–2.71)
Non-drinkers	GG	234/228		1.00	100/187		1.00
	GA	163/177	0.417	0.88(0.65–1.20)	75/138	0.388	0.84(0.56–1.25)
	AA	26/33	0.586	0.84(0.46–1.56)	14/28	0.851	0.93(0.45–1.93)
	GA + AA VS. GG		0.371	0.87(0.65–1.17)		0.387	0.85(0.58–1.24)
	AA VS. GA + GG		0.675	0.88(0.49–1.58)		0.963	1.02(0.50–2.08)
**pri-miR-365b rs121224**							
*H. pylori*^a^		n = 862 vs 862		n = 724 vs 729			
Negative	CC	79/118		1.00	68/99		1.00
	CG	160/304	0.402	0.88(0.65–1.19)	182/258	0.403	1.14(0.84–1.56)
	GG	118/199	0.492	1.14(0.79–1.64)	105/171	0.612	1.11(0.75–1.64)
	CG + GG VS. CC		0.713	0.95(0.72–1.25)		0.411	1.13(0.84–1.52)
	GG VS. CG + CC		0.235	1.22(0.88–1.67)		0.901	1.02(0.73–1.44)
Positive	CC	98/46		1.00	59/39		1.00
	CG	253/124	0.85	0.97(0.68–1.38)	194/104	0.806	0.95(0.64–1.42)
	GG	154/71	0.966	1.01(0.64–1.59)	116/58	0.262	0.74(0.44–1.25)
	CG + GG VS. CC		0.886	0.98(0.70–1.37)		0.565	0.90(0.61–1.31)
	GG VS. CG + CC		0.915	1.02(0.69–1.51)		0.263	0.77(0.49–1.21)
Smoking status^b^		n = 548 vs 586		n = 333 vs 500			
Smokers	CC	32/31		1.00	23/30		1.00
	CG	87/97	0.413	1.23(0.75–1.99)	81/95	0.273	1.34(0.80–2.24)
	GG	54/74	0.442	1.30(0.67–2.53)	44/74	0.688	1.16(0.57–2.36)
	CG + GG VS. CC		0.371	1.23(0.78–1.95)		0.303	1.29(0.79–2.12)
	GG VS. CG + CC		0.576	1.18(0.66–2.10)		0.954	0.98(0.52–1.85)
Non-smokers	CC	81/75		1.00	32/63		1.00
	CG	172/194	0.29	0.82(0.58–1.18)	100/150	0.402	1.21(0.77–1.90)
	GG	122/115	0.849	0.96(0.62–1.48)	53/88	0.496	0.82(0.45–1.47)
	CG + GG VS. CC		0.387	0.86(0.62–1.21)		0.699	1.09(0.71–1.67)
	GG VS. CG + CC		0.745	1.07(0.72–1.57)		0.210	0.73(0.44–1.20)
Drinking status^b^		n = 547 vs 585		n = 296 vs 499			
Drinkers	CC	25/21		1.00	19/20		1.00
	CG	64/81	0.583	1.18(0.66–2.09)	57/81	0.652	1.15(0.63–2.09)
	GG	35/45	0.19	1.69(0.77–3.70)	31/45	0.305	1.54(0.68–3.51)
	CG + GG VS. CC		0.369	1.29(0.74–2.23)		0.497	1.22(0.69–2.17)
	GG VS. CG + CC		0.29	1.43(0.74–2.76)		0.365	1.39(0.68–2.83)
Non-drinkers	CC	88/84		1.00	30/72		1.00
	CG	195/210	0.393	0.86(0.62–1.21)	104/164	0.267	1.28(0.83–1.97)
	GG	140/144	0.872	0.97(0.64–1.46)	55/117	0.308	0.74(0.41–1.32)
	CG + GG VS. CC		0.487	0.90(0.65–1.22)		0.617	1.11(0.74–1.68)
	GG VS. CG + CC		0.839	1.04(0.72–1.50)		0.087	0.645(0.39–1.07)
**pri-miR4795 rs1002765**							
*H. pylori*^a^		n = 862 vs 862		n = 724 vs 729			
Negative	GG	112/232		1.00	130/197		1.00
	GA	183/302	0.108	1.27(0.95–1.70)	167/259	0.982	1.00(0.74–1.34)
	AA	62/87	0.055	1.47(0.99–2.19)	58/72	0.387	1.20(0.79–1.82)
	GA + AA VS. GG		0.051	1.32(1.00–1.74)		0.770	1.04(0.79–1.38)
	AA VS. GA + GG		0.158	1.29(0.91–1.84)		0.307	1.22(0.84–1.77)
Positive	GG	192/75		1.00	142/65		1.00
	GA	233/118	0.143	0.77(0.54–1.09)	182/98	0.429	0.86(0.58–1.26)
	AA	80/48	0.129	0.70(0.45–1.11)	45/38	**0.050**	**0.58(0.34–1.00)**
	GA + AA VS. GG		0.085	0.75(0.54–1.04)		0.186	0.78(0.54–1.13)
	AA VS. GA + GG		0.293	0.81(0.54–1.21)		0.055	0.63(0.39–1.01)
Smoking status^b^		n = 548 vs 586		n = 333 vs 500			
Smokers	GG	53/73		1.00	60/73		1.00
	GA	86/105	0.517	1.18(0.72–1.93)	71/102	0.514	0.85(0.52–1.39)
	AA	34/24	0.073	1.93(0.94–3.96)	17/24	0.462	0.74(0.33–1.65)
	GA + AA VS. GG		0.279	1.29(0.81–2.06)		0.470	0.84(0.52–1.35)
	AA VS. GA + GG		0.087	1.70(0.93–3.14)		0.655	0.85(0.41–1.75)
Non-smokers	GG	139/130		1.00	62/98		1.00
	GA	182/190	0.705	0.94(0.66–1.32)	99/156	0.909	0.98(0.63–1.50)
	AA	54/64	0.247	0.76(0.47–1.22)	24/47	0.519	0.82(0.45–1.50)
	GA + AA VS. GG		0.463	0.89(0.64–1.23)		0.750	0.94(0.62–1.41)
	AA VS. GA + GG		0.271	0.78(0.51–1.21)		0.526	0.84(0.48–1.46)
Drinking status^b^		n = 547 vs 585		n = 296 vs 499			
Drinkers	GG	35/47		1.00	40/47		1.00
	GA	65/76	0.674	1.13(0.64–2.01)	51/75	0.328	0.75(0.42–1.34)
	AA	24/24	0.334	1.46(0.68–3.17)	16/24	0.607	0.81(0.35–1.84)
	GA + AA VS. GG		0.513	1.20(0.70–2.07)		0.347	0.77(0.44–1.33)
	AA VS. GA + GG		0.365	1.36(0.70–2.64)		0.935	0.97(0.47–2.02)
Non-drinkers	GG	156/156		1.00	61/124		1.00
	GA	203/218	0.979	1.00(0.73–1.39)	104/182	0.650	1.10(0.72–1.68)
	AA	64/64	0.657	0.90(0.57–1.43)	24/47	0.941	0.98(0.53–1.80)
	GA + AA VS. GG		0.893	0.98(0.72–1.33)		0.722	1.08(0.72–1.61)
	AA VS. GA + GG		0.639	0.91(0.60–1.37)		0.741	0.91(0.52–1.60)

Note: ^a^*P* values were adjusted by age and sex; ^b^*P* values were adjusted by age, sex and *H. pylori* infection status.

**Table 4:** The relationship between pri-let-7e rs8111742, pri-miR-365b rs121224, pri-miR-4795 rs1002765 and the risk of intestinal type and diffuse type gastric cancer.

Variables	Control group	Intestinal type gastric cancer	Diffuse type gastric cancer	Intestinal type gastric cancer vs. Control group		Diffuse type gastric cancer vs. Control group	
				*P* value	OR (95%CI)	*P* value	OR (95%CI)
**pri-let7e rs8111742**							
GG	389(53.4)	145(53.5)	185(50.3)		1.00		1.00
GA	285(39.1)	107(39.5)	146(39.7)	0.917	1.02(0.75–1.38)	0.566	1.08(0.83–1.42)
AA	55(7.5)	19(7.0)	37(10.1)	0.731	0.90(0.51–1.62)	0.161	1.40(0.88–2.23)
AA + GA vs. GG				0.985	1.00(0.75–1.34)	0.325	1.14(0.88–1.48)
AA vs. GA + GG				0.692	0.89(0.50–1.58)	0.173	1.37(0.87–2.15)
**pri-miR-365b rs121224**							
CC	138(18.9)	59(21.8)	58(15.8)		1.00		1.00
CG	362(49.7)	132(48.7)	199(54.1)	0.941	1.01(0.72–1.42)	0.456	1.12(0.83–1.50)
GG	229(31.4)	80(29.5)	111(30.2)	0.336	1.23(0.81–1.86)	0.477	0.87(0.59–1.29)
CG + GG vs. CC				0.665	1.07(0.78–1.47)	0.731	1.05(0.79–1.39)
GG vs. CG + CC				0.283	1.22(0.85–1.74)	0.232	0.81(0.57–1.14)
**pri-miR4795 rs1002765**							
GG	262(35.9)	101(37.3)	142(38.6)		1.00		1.00
GA	357(49.0)	124(45.8)	181(49.2)	0.499	0.90(0.65–1.23)	0.531	0.91(0.69–1.21)
AA	110(15.1)	46(17.0)	45(12.2)	0.721	1.08(0.70–1.68)	0.163	0.74(0.49–1.13)
AA + GA vs. GG				0.686	0.94(0.70–1.27)	0.318	0.87(0.67–1.14)
AA vs. GA + GG				0.509	1.14(0.77–1.70)	0.192	0.77(0.53–1.14)

**Table 5:** Stratified analysis of pri-let-7e rs8111742, pri-miR-365b rs121224, pri-miR-4795 rs1002765 and risk of intestinal type and diffuse type gastric cancer.

Variables	Genotype	Control group	Intestinal type gastric cancer	Diffuse type gastric cancer	Intestinal type gastric cancer vs. control group		Diffuse type gastric cancer vs. control group	
					*P*	OR(95%CI)	*P*	OR(95%CI)
**pri-let7e rs8111742**								
*H. pylori*^a^								
Negative	GG	278(52.7)	75(58.1)	95(52.8)		1.00		1.00
	GA	206(39.0)	49(38.0)	69(38.3)	0.573	0.89(0.60–1.33)	0.816	0.96(0.67–1.37)
	AA	44(8.3)	5(3.9)	16(8.9)	0.118	0.46(0.18–1.21)	0.621	1.17(0.63–2.16)
	GA + AA vs. GG				0.321	0.82(0.56–1.21)	0.966	0.99(0.71–1.39)
	AA vs. GA + GG				0.136	0.48(0.19–1.26)	0.587	1.18(0.65–2.15)
Positive	GG	111(55.2)	70(49.3)	90(47.9)		1.00		1.00
	GA	79(39.3)	58(40.8)	77(41.0)	0.454	1.19(0.75–1.89)	0.434	1.18(0.78–1.77)
	AA	11(5.5)	14(9.9)	21(11.2)	0.229	1.65(0.73–3.75)	0.057	2.01(0.98–4.12)
	GA + AA vs. GG				0.313	1.25(0.81–1.94)	0.177	1.30(0.89–1.91)
	AA vs. GA + GG				0.339	1.48(0.66–3.32)	0.059	1.95(0.97–3.92)
Smoking status^b^								
Smokers	GG	104(52.3)	39(53.4)	28(42.4)		1.00		1.00
	GA	78(39.2)	29(39.7)	32(48.5)	0.972	1.01(0.56–1.84)	0.113	1.65(0.89–3.06)
	AA	17(8.5)	5(6.8)	6(9.1)	0.666	0.77(0.24–2.51)	0.409	1.61(0.52–4.95)
	GA + AA vs. GG				0.914	0.97(0.54–1.74)	0.085	1.69(0.93–3.08)
	AA vs. GA + GG				0.612	0.74(0.23–2.39)	0.628	1.30(0.45–3.80)
Non-smokers	GG	159(52.8)	35(53.)	49(50.5)		1.00		1.00
	GA	122(40.5)	27(40.9)	39(40.2)	0.813	0.93(0.53–1.66)	0.636	0.89(0.54–1.45)
	AA	20(6.6)	4(6.1)	9(9.3)	0.911	0.94(0.29–2.98)	0.484	1.37(0.57–3.26)
	GA + AA vs. GG				0.795	0.93(0.53–1.62)	0.812	0.95(0.59–1.51)
	AA vs. GA + GG				0.903	0.93(0.30–2.89)	0.382	1.45(0.63–3.35)
Drinking status^b^								
Drinkers	GG	73(50.0)	20(50.0)	25(42.4)		1.00		1.00
	GA	63(43.2)	18(45.0)	28(47.5)	0.638	1.21(0.56–2.62)	0.254	1.46(0.76–2.81)
	AA	10(6.8)	2(5.0)	6(10.2)	0.565	0.59(0.10–3.53)	0.354	1.71(0.55–5.35)
	GA + AA vs. GG				0.743	1.14(0.53–2.42)	0.185	1.53(0.82–2.88)
	AA vs. GA + GG				0.557	0.59(0.10–3.40)	0.531	1.42(0.47–4.25)
Non-drinkers	GG	187(53.0)	35(53.0)	49(49.0)		1.00		1.00
	GA	138(39.1)	26(39.4)	42(42.0)	0.689	0.89(0.49–1.59)	0.838	0.95(0.59–1.54)
	AA	28(7.9)	5(7.6)	9(9.0)	0.877	0.92(0.32–2.66)	0.486	1.35(0.58–3.14)
	GA + AA vs. GG				0.654	0.88(0.50–1.54)	0.973	1.01(0.64–1.60)
	AA vs. GA + GG				0.941	0.96(0.34–2.75)	0.399	1.42(0.63–3.20)
**pri-miR-365b rs121224**								
*H. pylori*^a^								
Negative	CC	171(32.4)	36(27.9)	51(28.3)		1.00		1.00
	CG	258(48.9)	62(48.1)	96(53.3)	0.621	1.12(0.71–1.77)	0.250	1.26(0.85–1.85)
	GG	99(18.8)	31(24.0)	33(18.3)	0.144	0.50(0.87–2.57)_	0.699	1.10(0.67–1.82)
	CG + GG vs. CC				0.348	1.23(0.80–1.88)	0.314	1.21(0.84–1.75)
	GG vs. CG + CC				0.147	1.41(0.89–2.23)	0.837	0.96(0.62–1.47)
Positive	CC	58(28.9)	44(31.0)	60(31.9)		1.00		1.00
	CG	104(51.7)	70(49.3)	103(54.8)	0.663	0.90(0.54–1.47)	0.969	0.99(0.64–1.53)
	GG	39(19.4)	28(19.7)	25(13.3)	0.779	0.91(0.48–1.75)	0.157	0.65(0.36–1.18)
	CG + GG vs. CC				0.665	0.90(0.56–1.45)	0.617	0.90(0.59–1.36)
	GG vs. CG + CC				0.996	1.00(0.58–1.73)	0.121	0.66(0.39–1.12)
Smoking status^b^								
Smokers	CC	74(37.2)	20(27.4)	19(28.8)		1.00		1.00
	CG	95(47.7)	38(52.1)	39(259.1)	0.411	1.32(0.68–2.56)	0.340	1.39(0.71–2.70)
	GG	30(15.1)	15(20.5)	8(12.1)	0.475	1.33(0.61–2.87)	0.948	0.97(0.37–2.55)
	CG + GG vs. CC				0.328	1.37(0.73–2.58)	0.424	1.30(0.68–2.46)
	GG vs. CG + CC				0.512	1.28(0.61–2.69)	0.625	0.81(0.34–1.92)
Non-smokers	CC	88(29.2)	19(28.8)	28(28.9)		1.00		1.00
	CG	150(49.8)	30(45.5)	56(57.7)	0.956	1.02(0.53–1.95)		
	GG	63(20.9)	17(25.8)	13(13.4)	0.361	1.50(0.63–3.59)	0.317	0.68(0.32–1.44)
	CG + GG vs. CC				0.755	1.10(0.60–2.03)	0.742	1.09(0.65–1.82)
	GG vs. CG + CC				0.430	1.29(0.68–2.45)	0.109	0.58(0.30–1.13)
Drinking status^b^								
Drinkers	CC	45(30.8)	8(20.0)	21(35.6)		1.00		1.00
	CG	81(55.0)	21(52.5)	30(50.8)	0.288	1.69(0.64–4.46)	0.706	0.88(0.44–1.74)
	GG	20(13.7)	11(27.5)	8(13.6)	**0.029**	**3.76(1.15**–**12.34)**	0.756	0.85(0.32–2.31)
	CG + GG vs. CC				0.131	2.02(0.81–5.05)	0.688	0.87(0.45–1.69)
	GG vs. CG + CC				**0.045**	**2.52(1.02**–**6.23)**	0.833	0.91(0.37–2.23)
Non-drinkers	CC	117(33.1)	20(30.3)	26(26.0)		1.00		1.00
	CG	164(45.6)	30(45.5)	62(62.0)	0.978	0.99(0.52–1.88)	0.104	1.56(0.91–2.66)
	GG	72(20.4)	16(24.2)	12(12.0)	0.861	1.07(0.49–2.35)	0.407	0.72(0.34–1.56)
	CG + GG vs. CC				0.933	1.03(0.56–1.87)	0.302	1.31(0.78–2.19)
	GG vs. CG + CC				0.781	1.10(0.57–2.13)	0.073	0.54(0.27–1.06)
**pri-miR4795 rs1002765**								
*H. pylori*^a^								
Negative	GG	197(37.3)	49(38.0)	66(36.7)		1.00		1.00
	GA	259(49.1)	58(45.0)	86(47.8)	0.898	0.97(0.64–1.49)	0.882	1.03(0.71–1.48)
	AA	72(13.6)	22(17.1)	28(15.6)	0.422	1.27(0.71–2.24)	0.712	1.10(0.66–1.85)
	GA + AA vs. GG				0.850	1.04(0.70–1.55)	0.809	1.04(0.74–1.48)
	AA vs. GA + GG				0.319	1.31(0.77–2.20)	0.685	1.10(0.69–1.76)
Positive	GG	65(32.3)	52(36.6)	76(40.4)		1.00		1.00
	GA	98(48.8)	66(46.5)	95(50.5)	0.337	0.79(0.49–1.28)	0.295	0.80(0.53–1.22)
	AA	38(18.9)	24(16.9)	17(9.0)	0.504	0.80(0.41–1.56)	**0.005**	**0.39(0.20**–**0.75)**
	GA + AA vs. GG				0.317	0.79(0.50–1.25)	0.062	0.68(0.46–1.02)
	AA vs. GA + GG				0.771	0.92(0.52–1.63)	**0.005**	**0.43(0.24**–**0.78)**
Smoking status^b^								
Smokers	GG	73(36.7)	29(39.7)	28(42.4)		1.00		1.00
	GA	102(51.3)	32(43.8)	34(51.5)	0.492	0.80(0.43–1.51)	0.454	0.78(0.41–1.48)
	AA	24(12.1)	12(16.4)	4(6.1)	0.986	0.99(0.39–2.51)	0.057	0.29(0.08–1.04)
	GA + AA vs. GG				0.648	0.87(0.48–1.59)	0.258	0.70(0.38–1.30)
	AA vs. GA + GG				0.785	1.12(0.49–2.58)	0.153	0.43(0.14–1.37)
Non-smokers	GG	98(32.6)	23(34.8)	32(33.0)		1.00		1.00
	GA	156(51.8)	34(51.5)	52(53.6)	0.924	1.03(0.56–1.89)	0.692	1.11(0.66–1.87)
	AA	47(15.6)	9(13.6)	13(13.4)	0.58	0.78(0.33–1.86)	0.438	0.75(0.35–1.57)
	GA + AA vs. GG				0.889	0.96(0.54–1.71)	0.941	1.02(0.62–1.67)
	AA vs. GA + GG				0.496	0.76(0.35–1.66)	0.308	0.71(0.36–1.38)
Drinking status^b^								
Drinkers	GG	47(32.2)	9(22.5)	28(47.5)		1.00		1.00
	GA	75(51.4)	21(52.5)	26(44.1)	0.428	1.45(0.58–3.65)	0.070	0.53(0.27–1.05)
	AA	24(16.4)	10(25.0)	5(8.5)	0.187	2.19(0.68–7.03)	0.069	0.35(0.11–1.09)
	GA + AA vs. GG				0.270	1.65(0.68–3.99)	**0.032**	**0.49(0.25**–**0.94)**
	AA vs. GA + GG				0.274	1.68(0.66–4.26)	0.232	0.53(0.19–1.51)
Non-drinkers	GG	124(35.1)	24(36.4)	30(30.0)		1.00		1.00
	GA	182(51.6)	32(48.5)	58(58.0)	0.931	0.97(0.53–1.79)	0.308	1.31(0.78–2.20)
	AA	47(13.3)	10(15.2)	12(12.0)	0.857	0.92(0.39–3.19)	0.661	0.84(0.39–1.81)
	GA + AA vs. GG				0.900	0.96(0.54–1.72)	0.472	1.20(0.73–1.97)
	AA vs. GA + GG				0.844	0.93(0.43–1.99)	0.314	0.70(0.35–1.40)

Note: ^a^*P* values were adjusted by age and sex; ^b^*P* values were adjusted by age, sex and *H. pylori* infection status.

**Table 6:** Correlation between pri-let-7e rs8111742, pri-miR-365b rs121224, pri-miR-4795 rs1002765 polymorphism and clinical pathological parameters.

Clinical pathological parameters	Case number	Genotype			Dominant model *P*	Recessive model *P*
		wild type	hybrid type	mutation type		
**pri-let7e rs8111742**	357					
Age					0.113	0.802
≤60	168	73(43.5)	82(48.8)	13(7.7)		
>60	189	98(51.9)	75(39.7)	16(8.5)		
Gender					0.228	0.902
Male	255	117(45.9)	117(45.9)	21(8.2)		
Female	102	54(52.9)	40(39.2)	8(7.8)		
Borrmann type					0.850	0.564
Borrmann I–II	83	39(47.0)	36(43.4)	8(9.6)		
Borrmann III–IV	274	132(48.2)	121(44.2)	21(7.7)		
Lauren type					0.064	0.138
Intestinal type	120	66(55.0)	48(40.0)	6(5.0)		
Diffuse type	231	103(44.6)	106(45.9)	22(9.5)		
Non classified carcinoma	6	2(33.3)	3(50.0)	1(16.7)		
TNM staging					0.280	0.131
I–II	171	87(50.9)	74(43.3)	10(5.8)		
III–IV	186	84(45.2)	83(44.6)	19(10.2)		
Depth of invasion					0.989	0.652
T1 + T2	98	47(48.0)	42(42.9)	9(9.2)		
T3 + T4	259	124(47.9)	115(44.4)	20(7.7)		
Lymph node metastasis					0.325	0.121
Positive	216	98(45.4)	95(44.0)	23(10.6)		
Negative	141	73(51.8)	62(44.0)	6(4.3)		
**pri-miR-365b rs121224**	347					
Age					0.647	0.507
≤60	163	47(28.8)	85(52.1)	31(19.0)		
>60	184	49(26.6)	105(57.1)	30(16.3)		
Gender					0.193	0.809
Male	249	64(25.7)	142(57.0)	43(17.3)		
Female	98	32(32.7)	48(49.0)	18(18.4)		
Borrmann type					0.393	0.235
Borrmann I–II	83	26(31.3)	46(55.4)	11(13.3)		
Borrmann III–IV	264	70(26.5)	144(54.5)	50(18.9)		
Lauren type					0.329	**0.014**^b^
Intestinal type	118	36(30.5)	53(44.9)	29(24.6)		
Diffuse type	223	57(25.6)	135(60.5)	31(13.9)		
Non classified carcinoma	6					
TNM staging					0.961	**0.031**^c^
I–II	167	46(27.5)	84(50.3)	37(22.2)		
III–IV	180	50(27.8)	106(58.9)	24(13.3)		
Depth of invasion					0.976	0.809
T1 + T2	98	27(27.6)	53(54.1)	18(18.4)		
T3 + T4	249	69(27.7)	137(55.0)	43(17.3)		
Lymph node metastasis					0.237	0.624
Positive	210	56(26.7)	120(57.1)	34(16.2)		
Negative	137	40(29.2)	70(51.1)	27(19.7)		
**pri-mir4795 rs1002765**	357					
Age					0.170	0.240
≤60	168	65(38.7)	82(48.8)	21(12.5)		
>60	189	60(31.7)	97(51.3)	32(16.9)		
Gender					0.160	0.541
Male	255	95(37.3)	124(48.6)	36(14.1)		
Female	102	30(29.4)	55(53.9)	17(16.7)		
Borrmann type					0.064	0.345
Borrmann I–II	83	22(26.5)	46(55.4)	15(18.1)		
Borrmann III–IV	274	103(37.6)	133(48.5)	38(13.9)		
Lauren type					0.823	0.782
Intestinal type	120	43(35.8)	58(48.3)	19(15.8)		
Diffuse type	231	80(34.6)	117(50.6)	34(14.7)		
Non classified carcinoma	6	2(33.3)	4(66.6)	0(0.0)		
TNM staging					0.846	0.908
I–II	171	59(34.5)	87(50.9)	25(14.6)		
III–IV	186	66(35.5)	92(49.5)	28(15.1)		
Depth of invasion					0.565	0.880
T1 + T2	98	32(32.7)	51(52.0)	15(15.3)		
T3 + T4	259	93(35.9)	128(49.4)	38(14.7)		
Lymph node metastasis					0.753	0.916
Positive	216	75(34.7)	109(50.5)	32(14.8)		
Negative	141	50(35.5)	70(49.6)	21(14.9)		

Note: a, using chi square test; b, a multivariate regression analysis was used to correct for age and sex factors *P* = 0.014, OR = 1.42, 95%CI = 0.87–2.47; c, a multivariate regression analysis was used to correct for age and sex factors *P* = 0.031, OR = 1.37, 95%CI = 1.03–1.82.

**Table 7:** Relationship between miRNAs polymorphism and prognosis of gastric cancer in different subgroups.

Clinical pathological parameters	Genotype (death/total)			Dominant model		Recessive model	
	wild type	hybrid type	mutation type	*P*	HR	*P*	HR
**pri-let7e rs8111742**							
Age							
≤60	19/82	18/71	3/9	0.670	1.15(0.62–2.13)	0.389	1.51(0.59–3.86)
>60	29/100	16/72	2/15	0.854	0.95(0.54–1.67)	0.608	1.27(0.51–3.22)
Gender							
Male	36/131	25/109	1/14	0.902	0.97(0.59–1.59)	0.612	1.24(0.54–2.89)
Female	12/53	10/39	4/10	0.539	1.27(0.59–2.76)	0.380	1.61(0.56–4.69)
Borrmann type							
Borrmann I–II	13/37	10/31	0/5	0.140	0.53(0.23–1.23)	0.464	1.58(0.47–5.32)
Borrmann III–IV	35/128	23/99	5/18	0.230	1.35(0.83–2.20)	0.450	1.35(0.62–2.96)
Lauren type							
Intestinal type	14/69	10/53	2/7	0.512	0.75(0.32–1.76)	0.435	0.05(0.00–105.98)
Diffuse type	32/112	24/93	3/17	0.485	1.20(0.72–2.01)	0.079	1.84(0.93–3.62)
TNM staging							
I–II	20/91	11/75	0/10	0.052	0.22(0.05–1.01)	0.510	0.04(0.00–484.52)
II–IV	28/93	24/73	5/14	0.573	1.14(0.73–1.79)	0.298	1.42(0.73–2.77)
Depth of invasion	0/43	1/34	0/7	0.591	0.52(0.05–5.71)	0.653	NA
T1 + T2	32/102	25/87	5/13	0.715	1.08(0.71–1.65)	0.071	1.84(0.95–3.56)
T3 + T4							
Lymph node metastasis							
Positive	38/110	32/88	5/15	0.610	1.12(0.72–1.76)	0.593	1.20(0.62–2.33)
Negative	10/74	2/59	0/9	0.141	0.38(0.10–1.39)	0.611	NA
**pri-miR-365b rs121224**							
Age							
≤60	13/43	23/83	3/31	0.522	0.82(0.45–1.51)	0.066	0.33(0.10–1.08)
>60	15/48	26/104	6/30	0.473	0.79(0.41–1.51)	0.457	0.72(0.31–1.70)
Gender							
Male	20/63	36/142	6/43	0.246	0.73(0.43–1.24)	0.087	0.48(0.21–1.11)
Female	9/32	13/48	3/18	0.930	0.96(0.43–2.19)	0.448	0.63(0.19–2.10)
Borrmann type							
Borrmann I–II	10/25	11/39	2/9	0.172	0.56(0.25–1.28)	0.703	0.75(0.18–3.22)
Borrmann III–IV	18/59	37/129	7/47	0.686	0.90(0.53–1.53)	**0.042**	0.44(0.20–0.97)
Lauren type							
Intestinal type	9/38	15/59	2/30	0.626	0.82(0.36–1.85)	**0.031**	0.20(0.05–0.86)
Diffuse type	18/54	34/130	6/30	0.486	0.82(0.47–1.43)	0.577	0.79(0.34–1.83)
TNM staging							
I–II	13/52	15/83	3/34	0.065	0.34(0.11–1.07)	0.296	0.34(0.04–2.60)
III–IV	16/43	34/107	6/27	0.917	1.03(0.63–1.68)	0.458	0.76(0.36–1.58)
Depth of invasion							
T1 + T2	1/21	0/46	0/17	0.203	0.21(0.02–2.32)	0.607	NA
T3 + T4	17/46	37/109	7/37	0.428	0.83(0.53–1.31)	0.088	0.55(0.28–1.10)
Lymph node metastasis							
Positive	22/54	44/119	8/34	0.833	0.95(0.58–1.55)	0.164	0.59(0.29–1.24)
Negative	6/40	5/71	1/27	0.067	0.35(0.11–1.08)	0.346	0.37(0.05–2.89)
**pri-mir4795 rs1002765**							
Age							
≤60	14/64	21/79	5/19	0.952	1.02(0.53–1.95)	0.771	0.87(0.34–2.22)
>60	17/58	19/96	11/32	0.258	0.71(0.39–1.28)	0.351	1.38(0.70–2.70)
Gender							
Male	23/95	28/123	11/35	0.528	0.85(0.51–1.42)	0.531	1.23(0.64–2.36)
Female	42246	13/55	5/17	0.594	0.80(0.35–1.84)	0.963	0.98(0.37–2.60)
Borrmann type							
Borrmann I–II	7/17	10/41	6/15	0.711	0.91(0.55–1.50)	0.337	1.58(0.62–4.00)
Borrmann III–IV	23/91	30/120	10/33	0.509	0.74(0.31–1.80)	0.936	1.03(0.52–2.02)
Lauren type							
Intestinal type	9/45	13/64	4/19	0.693	0.85(0.37–1.92)	0.851	0.90(0.31–2.63)
Diffuse type	22/79	25/110	12/33	0.279	0.75(0.44–1.27)	0.411	1.30(0.69–2.45)
TNM staging							
I–II	7/55	16/92	8/28	0.254	2.42(0.53–11.05)	0.417	1.72(0.46–6.38)
III–IV	24/70	25/86	8/24	0.078	0.66(0.41–1.05)	0.897	1.04(0.57–1.89)
Depth of invasion							
T1 + T2	1/29	0/44	0/11	0.211	0.22(0.02–2.38)	0.626	NA
T3 + T4	23/77	30/98	9/26	0.598	0.89(0.57–1.39)	0.507	1.20(0.70–2.07)
Lymph node metastasis							
Positive	29/75	33/107	13/31	0.086	0.67(0.42–1.06)	0.734	1.11(0.61–2.01)
Negative	2/50	7/70	3/21	0.232	2.53(0.55–11.54)	0.503	1.57(0.42–5.81)

HR, hazard rate; NA, not avalable.

## References

[b1] Bottoni A. . miR-15a and miR-16-1 down-regulation in pituitary adenomas. J Cell Physiol. 204, 280–285 (2005). 15648093 10.1002/jcp.20282

[b2] Zhang L. S. . Association between pri-miR-218 polymorphism and risk of hepatocellular carcinoma in a Han Chinese population. DNA Cell Biol. 31, 761–765 (2012). 22011248 10.1089/dna.2011.1326

[b3] Bensen J. T. . Association of germline microRNA SNPs in pre-miRNA flanking region and breast cancer risk and survival: the Carolina Breast Cancer Study. Cancer Causes Control 24, 1099–1109 (2013). 23526039 10.1007/s10552-013-0187-z PMC3804004

[b4] Xing J. . Genetic polymorphisms in pre-microRNA genes as prognostic markers of colorectal cancer. Cancer Epidemiol Biomarkers Prev. 21, 217–227 (2012). 22028396 10.1158/1055-9965.EPI-11-0624 PMC3253954

[b5] Xu Q. . A new polymorphism biomarker rs629367 associated with increased risk and poor survival of gastric cancer in chinese by up-regulated miRNA-let-7a expression. PLoS One 9, e95249 (2014). 24760009 10.1371/journal.pone.0095249 PMC3997364

[b6] Kogo R. . Clinical significance of miR-146a in gastric cancer cases. Clin Cancer Res. 17, 4277–4284 (2011). 21632853 10.1158/1078-0432.CCR-10-2866

[b7] Hu Z. . Genetic variants of miRNA sequences and non-small cell lung cancer survival. J Clin Invest. 118, 2600–2608 (2008). 18521189 10.1172/JCI34934 PMC2402113

[b8] Yoon K. A. . The prognostic impact of microRNA sequence polymorphisms on the recurrence of patients with completely resected non-small cell lung cancer. J Thorac Cardiovasc Surg. 144, 794–807 (2012). 22818121 10.1016/j.jtcvs.2012.06.030

[b9] Permuth-Wey J. . A functional polymorphism in the pre-miR-146a gene is associated with risk and prognosis in adult glioma. J Neurooncol. 105, 639–646 (2011). 21744077 10.1007/s11060-011-0634-1 PMC3217154

[b10] Khvorova A., Reynolds A. & Jayasena S. D. Functional siRNAs and miRNAs exhibit strand bias. Cell 115, 209–216 (2003). 14567918 10.1016/s0092-8674(03)00801-8

[b11] Ning P. F., Liu H. J. & Yuan Y. Dynamic expression of pepsinogen C in gastric cancer, precancerous lesions and Helicobacter pylori associated gastric diseases. World J Gastroenterol. 11, 2545–2548 (2005). 15849808 10.3748/wjg.v11.i17.2545 PMC4305740

[b12] Tu H. . Temporal changes in serum biomarkers and risk for progression of gastric precancerous lesions: a longitudinal study. Int J Cancer. 136, 425–434 (2015). 24895149 10.1002/ijc.29005 PMC4354768

[b13] Sobin L. H. & Compton C. C. TNM seventh edition: what’s new, what’s changed: communication from the International Union Against Cancer and the American Joint Committee on Cancer. Cancer 116, 5336–5339 (2010). 20665503 10.1002/cncr.25537

[b14] Lauren P. The two histological main types of gastric carcinoma: diffuse and so-called intestinal-type carcinoma. An attempt at a histo-clinical classification. Acta Pathol Microbiol Scand. 64, 31–49 (1965). 14320675 10.1111/apm.1965.64.1.31

[b15] Xu Q., Liu J. W. & Yuan Y. Comprehensive assessment of the association between miRNA polymorphisms and gastric cancer risk. Mutat Res Rev Mutat Res. 763, 148–160 (2015). 25795117 10.1016/j.mrrev.2014.09.004

[b16] Zhou L. . microRNA-365-targeted nuclear factor I/B transcriptionally represses cyclin-dependent kinase 6 and 4 to inhibit the progression of cutaneous squamous cell carcinoma. Int J Biochem Cell Biol. 65, 182–191 (2015). 26072217 10.1016/j.biocel.2015.06.009

[b17] Kang S. M., Lee H. J. & Cho J. Y. MicroRNA-365 regulates NKX2-1, a key mediator of lung cancer. Cancer Lett. 335, 487–494 (2013). 23507558 10.1016/j.canlet.2013.03.006

[b18] Qi J. . MiR-365 regulates lung cancer and developmental gene thyroid transcription factor 1. Cell Cycle. 11, 177–186 (2012). 22185756 10.4161/cc.11.1.18576 PMC3272236

[b19] Zhou M. . A novel onco-miR-365 induces cutaneous squamous cell carcinoma. Carcinogenesis 34, 1653–1659 (2013). 23514750 10.1093/carcin/bgt097 PMC3697891

[b20] Chen Z. . Prognostic significance and anti-proliferation effect of microRNA-365 in hepatocellular carcinoma. Int J Clin Exp Pathol. 8, 1705–1711 (2015). 25973057 PMC4396306

[b21] Xiong X. D. . MicroRNA transcriptome analysis identifies miR-365 as a novel negative regulator of cell proliferation in Zmpste24-deficient mouse embryonic fibroblasts. Mutat Res. 777, 69–78 (2015). 25983189 10.1016/j.mrfmmm.2015.04.010 PMC4654172

[b22] Xu Z. . miR-365, a novel negative regulator of interleukin-6 gene expression, is cooperatively regulated by Sp1 and NF-kappaB. J Biol Chem. 286, 21401–21412 (2011). 21518763 10.1074/jbc.M110.198630 PMC3122200

[b23] Nie J. . microRNA-365, down-regulated in colon cancer, inhibits cell cycle progression and promotes apoptosis of colon cancer cells by probably targeting Cyclin D1 and Bcl-2. Carcinogenesis 33, 220–225 (2012). 22072615 10.1093/carcin/bgr245

[b24] Qin B. . MicroRNAs expression in ox-LDL treated HUVECs: MiR-365 modulates apoptosis and Bcl-2 expression. Biochem Biophys Res Commun. 410, 127–133 (2011). 21640710 10.1016/j.bbrc.2011.05.118

[b25] Gastaldi C. . miR-193b/365a cluster controls progression of epidermal squamous cell carcinoma. Carcinogenesis 35, 1110–1120 (2014). 24374827 10.1093/carcin/bgt490

[b26] Zhou M. . miR-365 promotes cutaneous squamous cell carcinoma (CSCC) through targeting nuclear factor I/B (NFIB). PLoS One 9, e100620 (2014). 24949940 10.1371/journal.pone.0100620 PMC4065106

[b27] Guo S. L. . Akt-p53-miR-365-cyclin D1/cdc25A axis contributes to gastric tumorigenesis induced by PTEN deficiency. Nat Commun. 4, 2544 (2013). 24149576 10.1038/ncomms3544 PMC3826643

[b28] Shearn C. T. . Increased carbonylation of the lipid phosphatase PTEN contributes to Akt2 activation in a murine model of early alcohol-induced steatosis. Free Radic Biol Med. 65, 680–692 (2013). 23872024 10.1016/j.freeradbiomed.2013.07.011 PMC3859727

[b29] He C. Y. . PGC TagSNP and its interaction with H. pylori and relation with gene expression in susceptibility to gastric carcinogenesis. PLoS One 9, e115955 (2014). 25551587 10.1371/journal.pone.0115955 PMC4281127

[b30] He C. . Polymorphic rs9471643 and rs6458238 upregulate PGC transcription and protein expression in overdominant or dominant models. Mol Carcinog. 55, 586–599 (2016). 25857852 10.1002/mc.22305

[b31] He C. . SNP interactions of Helicobacter pylori-related host genes PGC, PTPN11, IL1B, and TLR4 in susceptibility to gastric carcinogenesis. Oncotarget 6, 19017–19026 (2015). 26158864 10.18632/oncotarget.4231 PMC4662472

[b32] Moore J. H. & Williams S. M. New strategies for identifying gene-gene interactions in hypertension. Ann Med. 34, 88–95 (2002). 12108579 10.1080/07853890252953473

[b33] Cordell H. J. Detecting gene-gene interactions that underlie human diseases. Nat Rev Genet. 10, 392–404 (2009). 19434077 10.1038/nrg2579 PMC2872761

[b34] Moore J. H. The ubiquitous nature of epistasis in determining susceptibility to common human diseases. Hum Hered. 56, 73–82 (2003). 14614241 10.1159/000073735

[b35] Sapkota Y. . Assessing SNP-SNP interactions among DNA repair, modification and metabolism related pathway genes in breast cancer susceptibility. PLoS One 8, e64896 (2014). 23755158 10.1371/journal.pone.0064896 PMC3670937

[b36] Xu Q. . SNP-SNP interactions of three new pri-miRNAs with the target gene PGC and multidimensional analysis of H. pylori in the gastric cancer/atrophic gastritis risk in a Chinese population. Oncotarget (2016). 10.18632/oncotarget.8057 PMC5029657 26988755

[b37] Liu W. J. . Expression of serum let-7c, let-7i, and let-7f microRNA with its target gene, pepsinogen C, in gastric cancer and precancerous disease. Tumour biology: the journal of the International Society for Oncodevelopmental Biology and Medicine 36, 3337–3343 (2015). 25549793 10.1007/s13277-014-2967-9

[b38] Sun Q. . Hsa-mir-27a genetic variant contributes to gastric cancer susceptibility through affecting miR-27a and target gene expression. Cancer science 101, 2241–2247 (2010). 20666778 10.1111/j.1349-7006.2010.01667.x PMC11159034

[b39] Tong N. . Pri-miR-34b/c rs4938723 polymorphism contributes to acute lymphoblastic leukemia susceptibility in Chinese children. Leukemia & lymphoma 57, 1436–1441 (2016). 26380954 10.3109/10428194.2015.1092528

[b40] Zhang S. . A potentially functional polymorphism in the promoter region of miR-34b/c is associated with renal cell cancer risk in a Chinese population. Mutagenesis 29, 149–154 (2014). 24503183 10.1093/mutage/geu001

[b41] Dixon M. F., Genta R. M., Yardley J. H. & Correa P. Classification and grading of gastritis. The updated Sydney System. International Workshop on the Histopathology of Gastritis, Houston 1994. Am J Surg Pathol. 20, 1161–1181 (1996). 8827022 10.1097/00000478-199610000-00001

[b42] Stolte M. & Meining A. The updated Sydney system: classification and grading of gastritis as the basis of diagnosis and treatment. Can J Gastroenterol 15, 591–598 (2001). 11573102 10.1155/2001/367832

[b43] Gong Y. H., Sun L. P., Jin S. G. & Yuan Y. Comparative study of serology and histology based detection of Helicobacter pylori infections: a large population-based study of 7,241 subjects from China. Eur J Clin Microbiol Infect Dis. 29, 907–911 (2010). 20440530 10.1007/s10096-010-0944-9

